# Visits to Private Asylums

**Published:** 1903-09-19

**Authors:** 


### VISITS TO PRIVATE ASYLUMS.

By OUR RAMBLING REPORTER.

### CLARENCE LODGE, CLAPHAM PARK, S.W.

FROM London Bridge this asylum may be reached by electric tramcar to Clapham Common, or from Victoria by train to Clapham Road, whence it is distant ten or twelve minutes' walk. The house is comparatively modern, being only about 30 years old. It is surrounded by three and a-half acres of land, and considering its short distance from London and its accessibility it is wonderfully secluded, and not overlooked on any side. The grounds are laid out in lawns, flower borders, shrubberies and orchards, and these are arranged so that a long, pleasant ramble may be had through the paths with an opportunity of looking in at the greenhouses and vineries. The house and its adjuncts are distinctly of the private mansion type, with nothing savouring of the institution about them, unless it be the presence of a fire-escape staircase. From the admirable way in which the bedrooms are arranged this was hardly an essential addition, but in the event of fire, it would render the escape of the patients absolutely certain. The asylum is licensed for the reception of 12 ladies; and the proprietress, who is a Graduate in Arts of the London University, has had much experience in the treatment of patients. The sitting-rooms and bedrooms are exceptionally bright-looking and airy, and from many of them nice views of the neighbourhood can be had. Some of the ladies dine privately in their own apartments, and others have late dinner with the proprietress. It is evident that the patients are treated with much consideration and kindness, and that they have considerable freedom. The staff of nurses is amply sufficient numerically, and, further, it is the aim of the proprietress to engage no one as a nurse who is not a lady, and thus the staff is brought into greater sympathy with the patients than would otherwise be likely. The result of all this is a wonderful amount of contentment on the part of the patients; and if there be any agitators who still clamour for the abolition of private asylums they could hardly do better than visit Clarence Lodge. This visit would convince them that such abolition, even if practicable, would be a real grievance and loss to the insane themselves. Personally we should like to see these smaller licensed houses increased in number, always provided that they were retained as private houses and not made into institutions with institutional formalities; and by "smaller" we mean those containing under 15 patients, that number being, we think, about the highest which can be managed satisfactorily in an asylum of this type, except under exceptional circumstances. Patients are admitted to Clarence Lodge at the rate of three and a-half guineas a week and upwards, according to the accommodation required.

